# Strategies to Increase Response Rate and Reduce Nonresponse Bias in Population Health Research: Analysis of a Series of Randomized Controlled Experiments during a Large COVID-19 Study

**DOI:** 10.2196/60022

**Published:** 2025-01-09

**Authors:** Christina J Atchison, Nicholas Gilby, Galini Pantelidou, Sam Clemens, Kevin Pickering, Marc Chadeau-Hyam, Deborah Ashby, Wendy S Barclay, Graham S Cooke, Ara Darzi, Steven Riley, Christl A Donnelly, Helen Ward, Paul Elliott

**Affiliations:** 1 School of Public Health, Imperial College London, London, United Kingdom; 2 Ipsos, London, United Kingdom; 3 Department of Infectious Disease, Imperial College London, Norfolk Place, London, United Kingdom; 4 Institute of Global Health Innovation, Imperial College London, South Kensington Campus, London, United Kingdom; 5 Department of Statistics, University of Oxford, Oxford, United Kingdom

**Keywords:** study recruitment, response rate, population-based research, COVID-19, SARS-CoV-2, web-based questionnaires

## Abstract

**Background:**

High response rates are needed in population-based studies, as nonresponse reduces effective sample size and bias affects accuracy and decreases the generalizability of the study findings.

**Objective:**

We tested different strategies to improve response rate and reduce nonresponse bias in a national population–based COVID-19 surveillance program in England, United Kingdom.

**Methods:**

Over 19 rounds, a random sample of individuals aged 5 years and older from the general population in England were invited by mail to complete a web-based questionnaire and return a swab for SARS-CoV-2 testing. We carried out several nested randomized controlled experiments to measure the impact on response rates of different interventions, including (1) variations in invitation and reminder letters and SMS text messages and (2) the offer of a conditional monetary incentive to return a swab, reporting absolute changes in response and relative response rate (95% CIs).

**Results:**

Monetary incentives increased the response rate (completed swabs returned as a proportion of the number of individuals invited) across all age groups, sex at birth, and area deprivation with the biggest increase among the lowest responders, namely teenagers and young adults and those living in more deprived areas. With no monetary incentive, the response rate was 3.4% in participants aged 18‐22 years, increasing to 8.1% with a £10 (US $12.5) incentive, 11.9% with £20 (US $25.0), and 18.2% with £30 (US $37.5) (relative response rate 2.4 [95% CI 2.0-2.9], 3.5 [95% CI 3.0-4.2], and 5.4 [95% CI 4.4-6.7], respectively). Nonmonetary strategies had a modest, if any, impact on response rate. The largest effect was observed for sending an additional swab reminder (SMS text message or email). For example, those receiving an additional SMS text message were more likely to return a completed swab compared to those receiving the standard email-SMS approach, 73.3% versus 70.2%: percentage difference 3.1% (95% CI 2.2%-4.0%).

**Conclusions:**

Conditional monetary incentives improved response rates to a web-based survey, which required the return of a swab test, particularly for younger age groups. Used in a selective way, incentives may be an effective strategy for improving sample response and representativeness in population-based studies.

## Introduction

In population-based studies, a high response rate from a representative sample may reduce nonparticipation bias, increase the generalizability, and improve the accuracy of study estimates [1]. However, achieving this goal is challenging, both due to the difficulty in contacting and then engaging eligible participants [2]. For example, UK Biobank, a population-based cohort study with stored biological samples from half a million participants aged 40‐69 years in the United Kingdom, achieved an overall response rate of 5.5% [3], which was lower in men, younger people, and those living in more deprived areas [4]. The impact of nonresponse and nonrepresentativeness on the generalizability of disease prevalence and incidence rates in the UK Biobank has been widely debated [5,6].

It is important to address low or falling response rates to reduce the likelihood of systematic biases that may affect study estimates [7]. While weighting is commonly applied to correct for differential participation, it may fail to correct bias if the responders in a particular subgroup of the population are not representative of that subgroup as a whole. Furthermore, weighting to correct for observed biases worsens precision (reducing the effective sample size) [8].

Systematic reviews that have evaluated interventions to increase response rates in surveys have concluded that monetary incentives are more effective than nonmonetary incentives [9-11], although findings were inconsistent concerning web-based surveys in educational research [12]. Some studies have found incentives can increase response among under-represented sociodemographic groups, such as those with low incomes, those with low education, single parents, and minority ethnic groups, potentially reducing nonresponse bias [13], while others show mixed results [9].

Other strategies that have been shown to improve response rates in surveys have included the use of SMS text message reminders to enhance the contact method of letters and emails [14,15], using alternative motivational statements in invitation letters [16], and changing the font color of text [17]. In a United Kingdom–based study investigating the effects of augmenting the contact strategy of letters and emails with SMS text messages for a web questionnaire, the findings indicated that SMS text messages did not help to significantly increase response rates overall, although some subgroups benefited from them, such as younger panel members and those with an irregular response pattern [15].

The Real-time Assessment of Community Transmission-1 (REACT-1) study was one of the largest population surveillance studies in the world. Across 19 rounds between May 1, 2020 and March 31, 2022, it provided timely prevalence estimates of SARS-CoV-2, the virus that causes COVID-19, from random cross-sectional samples of the population in England [18,19].

Response rate varied between 11.7% and 30.5% and, like in many population surveys, varied across demographic groups [19]. For financial reasons, we could not issue more than 845,000 invitation letters by mail, so we could only achieve the minimum desired sample size adopted from round 12 (May 20 to June 7, 2021) of 100,000 by improving response [18]. The observed nonresponse biases meant REACT-1 was under-representing groups with lower vaccination rates and where COVID-19 prevalence was highest; thus, we were likely underestimating the true population prevalence despite our attempts to correct for such biases by use of weighting on known demographic variables [20,21]. Here we present results of experiments nested within the REACT-1 study to test the effectiveness of different strategies to increase response rates and participation of groups with a lower propensity to take part.

## Methods

### The REACT-1 Study

Methods for the study, including sample size calculations, are described in detail elsewhere [18,19]. In summary, at approximately monthly intervals, between 395,020 and 841,227 people were sent personalized invitations by mail to take part. For children (5-17 years old), the invitation was sent to or via the parents or guardians. Individuals aged 5 years and older were randomly sampled from the National Health Service (NHS) list of patients in England (with near-universal population coverage) across all 316 Lower Tier Local Authorities [18,19]. This list includes the name, address, date of birth, and sex of everyone registered with a general practitioner in England. Invitees who registered (most digitally, some by telephone) for the study received a kit by mail with instructions on how to take a throat and nose swab and send it for SARS-CoV-2 testing using reverse transcriptase polymerase chain reaction (rt-PCR). Swabs were transferred to laboratories for processing, initially being picked up by courier with cold chain capacity (rounds 1-13 and part of 14) or sent by priority mail (part of round 14 and subsequent rounds). Participants were also asked to complete a self-administered web-based or telephone questionnaire [18,19].

**Figure 1.**
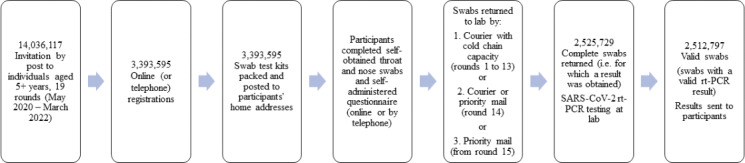
REACT-1 study process over 19 rounds of data collection: England, May 1, 2020 to March 31, 2022. Overall, across 19 rounds, we report the number of invitations sent, the number of participants registered, the number of swab test kits sent out, the number of completed swabs returned (ie, for which a laboratory result was obtained) and the number of valid swabs (swabs with a valid rt-PCR result). REACT-1: REal-time Assessment of Community Transmission-1; rt-PCR: reverse transcriptase Polymerase Chain Reaction; SARS-CoV-2: Severe Acute Respiratory Syndrome CoronaVirus 2.

Over the 19 study rounds, we sent out 14,036,117 invitations, 3,393,595 registrations were made, and 2,525,729 completed swabs were returned (ie, for which a laboratory result was obtained) (Figure 1). Of these swabs, 2,512,797 (99.5% of completed swabs returned) were considered valid for analysis in REACT-1 (swabs with a valid rt-PCR result) [19]. A swab with a valid rt-PCR result was a swab for which a “cycle threshold” (Ct) value could be obtained. Therefore, not all swabs tested by the lab were considered valid. Overall, 12,932 (0.5% of completed swabs returned) were considered invalid and rejected. Reasons included inadequate sample volume, contamination during sample collection, inappropriate sample storage, or inappropriate sample transportation. All analyses in this paper are based on completed swabs returned (ie, for which a laboratory result was obtained, n=2,525,729), thus including swabs for which a Ct value could not be obtained but excluding swabs returned unused.

All the experiments carried out to improve response rate were randomized trials, enabling an unbiased assessment of the impact of the changed survey procedure compared to a control group. Due to funding constraints, initial experiments focused on approaches which would not materially affect the survey budget, before turning to an experiment with monetary incentives.

### Swab Reminder and Tailored Letter or SMS Experiments

In each round of REACT-1, those registering for a swab test were, where necessary, sent at least one reminder to complete the swab test and return it, to maximize the number of swabs returned. In round 3 (July 24 to August 11, 2020) we conducted an experiment to establish the optimal use of email and SMS text message swab return reminders, with participants randomly allocated to the experimental conditions (Table 1).

**Table 1.** Round 3 swab reminder experimental conditions, England, July 24 to August 11, 2020.

Condition	Reminder on day 4 after swab test kit received	Reminder on day 6 after swab test kit received	Reminder on day 8 after swab test kit received	Sample, n
Control group	Email(SMS if no email address)	SMS	None	11,194
Experimental group A	SMS	Email(SMS if no email address)	None	11,154
Experimental group B	Email(SMS if no email address)	SMS	Email(SMS if no email address)	96,337
Experimental group C	SMS	Email(SMS if no email address)	SMS	96,305

The tailored letters or SMS experiments are summarized in Table 2. Further details are available in Multimedia Appendix 1. The experiments tested whether it was possible to increase participation by different types of conditions (Textbox 1).

**Table 2.** Rounds 9, 11, and 12 registration invitation letter experimental conditions and rounds 10 and 12 SMS registration reminder experimental conditions, England, February 4 to June 7, 2021.

Age and letter or SMS type	Additional content for experiment (actual additional content used in bold text)	Sample, n
Round 9^a^ (≥70 years)		
Standard invitation letter Adult	None	37,037
Experiment invitation letter A	“**It is still important to take part in this study if you have received a vaccination from COVID-19 or expect to be vaccinated in the near future**. Your participation will help DHSC assess the impact of the vaccines on COVID-19 infection rates.”As well as a new sub-heading “**COVID-19 Testing Study: Take part to help measure COVID-19 infection rates among those aged 70 and over**.”	37,037
Experiment invitation letter B	As per Experiment Letter A with additional line “**Older people are a vulnerable group, so we need your help to monitor prevalence**. It is still important to take part in this study if you have received a vaccination from COVID-19 or expect to be vaccinated in the near future.”	37,036
Round 9 (5‐12 years)		
Standard invitation letter Child (addressed to parent)	None	24,009
Experiment invitation letter C	**“We need to know how many children and young people have COVID-19, and how easily the new variant spreads amongst them.”**	24,009
Experiment invitation letter D	As per Experiment Letter C with new sub-heading **“COVID-19 Testing Study: Take part to help measure how easily COVID-19 spreads among children and young people.”**	24,008
Round 9 (all)		
Standard registration reminder letter	Blue text used	306,012
Experiment registration reminder letter E	Red text used	305,041
Round 11^b^ (≥18 years)		
Standard invitation reminder letter	None	178,828
Experiment invitation reminder letter A	New content asking participants to take a test to help prevent the spread of COVID-19 and explaining that taking part would help the Government work out the best way to manage the pandemic. Also mentioned testing for new variants, that the study compared people who had been vaccinated with those who had not, and that taking part would help inform the vaccine strategy and help to avoid lockdowns.	178,809
Round 12^c^ (all)		
Standard invitation final reminder letter	Double-sided	169,845
Shorter invitation final reminder letter	Single-sided	342,191
Round 10^d^ (all)		
Standard SMS first reminder	Unchanged**“The study is closing soon, please register by 18 March if you want to take part.”**	50,000
Experiment first SMS reminder	New SMS content“**Taking part will help inform decisions about the best time to lift restrictions**.”	430,283
Round 11 (all)		
Standard SMS second reminder	Unchanged“**The study is closing soon, please register by 3pm on 22 April if you want to take part**.”	127,028
Experiment second SMS reminder	New SMS content“**Taking part will help monitor infection rates and new variants of the virus**.”	127,028
Round 12 (all)		
Standard SMS first reminder	Unchanged“**Taking part will help inform decisions about the best time to lift restrictions**.”	321,042
Experiment first SMS reminder	New SMS Content**“Taking part will help monitor infection rates and new variants of the virus.”**	155,683
Standard SMS second reminder	Unchanged**“Taking part will help monitor infection rates and new variants of the virus.”**	272,836
Experiment second SMS reminder	New SMS Content**“Last chance to help monitor variants in your area.”**	136,026

a Round 9 (Feb 4-23, 2021)

b Round 11 (Apr 15 to May 3, 2021)

c Round 12 (May 20 to Jun 7, 2021)

d Round 10 (Mar 11-30, 2021)

Textbox 1. **Types of conditions tested in the tailored letters or SMS experiments.**

- Using additional content in the invitation letter, tailored for the oldest and youngest age groups.
- Using color, additional content, and varying the length of the reminder letter.
- Using additional content in the SMS reminder.

### Incentives Experiment

In round 15 (October 19 to Nov 5, 2021), conditional incentives (£10 [US $12.5], £20 [US $25.0], or £30 [US $37.5] gift vouchers for returning a completed swab test) were tested in a randomized controlled trial for all age groups except 5‐ to 12-year-olds.

The process for obtaining consent in REACT-1 for children was undertaken differently based on participant age at the time of the invitation [18]. For 5‐ to 12-year-olds, the parent or guardian was contacted via letter and asked to consent on behalf of the child. Therefore, we did not include the 5‐ to 12-year-olds in the trial, as the sampled child would not be making the decision to take the swab test, and their parent would be incentivized, raising ethical and reputational concerns. For 13‐ to 17-year-olds, the parent or guardian received a letter addressed to them, asking them to pass on an enclosed invitation letter addressed to their sampled child if they agreed for their child to take part in the study. As such, children aged 13‐17 years were able to decide whether to consent to the study and take the swab test. In addition, those aged 13 to 15 years were asked at registration to confirm the name of the parent or guardian who had given them permission to take part. This was not required for those aged 16‐17 years, as in UK health research, the Health Research Authority states that young people over 16 are presumed capable of giving consent on their own behalf [22].

Participants were randomly allocated to experimental and control groups: (1) £10 (US $12.5) conditional incentive (n=10,900), (2) £20 (US $25.0) conditional incentive (n=10,900), (3) £30 (US $37.5) conditional incentive (only for 18‐ to 32-year-olds) (n=1750), and (4) control group (n=23,500). Further details of the sample size calculations are available in Multimedia Appendix 1.

The £30 (US $37.5) incentive was limited to the 18‐ to 32-year-olds because the response rate in REACT-1 was lowest among this age group. Also, there is evidence that incentives can be more effective among younger age groups [23]. Those in this age group were of particular interest as they were less likely to be vaccinated, had more social contact (and therefore were more likely to be at risk of infection), and had been particularly impacted by the pandemic (in terms of well-being, education, and employment) [21,24]. It was decided to test offering a larger (£30 [US $37.5]) incentive to this age group (and not the other age groups) to overcome their higher reluctance to take part and to better represent this group in the achieved sample. Based on the same rationale, we oversampled younger age groups to maximize the statistical power we had to detect an increase in the response rate due to the use of incentives among these groups.

The primary outcome was overall swab response rate, ie, the number of completed swabs returned (referred to as swabs returned forthwith) as a proportion of the number of invitations sent. For those invited, we knew age, sex at birth, and score from an area-level index of multiple deprivation, the Index of Multiple Deprivation (IMD) 2019 [25]. Participants were classified by quintiles of the deprivation score based on their residential postcode.

We used COVID-19 vaccination status (the proportion who had received at least one vaccine dose) as a proxy for attitudes to health behaviors and health care access, hypothesizing that REACT-1 responders would be more likely to be vaccinated than those who did not, indicating a responder bias. Thus, the difference in vaccination status at registration between the experimental and control groups was used as a crude indicator of how incentives might improve response rates in individuals less likely to participate in research, beyond sociodemographic characteristics. We also compared the COVID-19 vaccination status of those who returned a swab with the achieved population vaccination rate for that age group as a whole. To obtain information on dates of received COVID-19 vaccine doses, participant study data were linked to their NHS records from NHS Digital (now NHS England) on COVID-19 vaccination events [26] using their unique NHS number and other personal identifiers. This was only possible for study participants who had consented to data linkage. The source of vaccination data for the population vaccination rates was the NHS National Immunization Management System [27].

### Ethical Considerations

The study was ethically approved by the South Central-Berkshire B Research Ethics Committee (IRAS ID: 283787). Participants provided informed consent when they registered for the study, and all data were handled securely in accordance with a detailed privacy notice. Collected data were deidentified; the data used in this study were anonymous and did not contain any personally identifiable information. Participants had the ability to opt out anytime during the research period. The study did not provide any specific compensation other than the monetary gift vouchers for returning a completed swab test as set out in the study’s incentives experiment described above.

### Statistical Analysis

Data analysis was conducted using IBM SPSS Statistics (version 28). As the incentives experiment was skewed toward younger age groups, swab response rates for sex at birth and area deprivation (IMD) were calculated with age-standardized weighting using 2021-based population estimates for England [28]. The percentage point difference (95% CI) and independent 2-tailed *t*-tests were used to show the absolute difference in swab response rates between the experimental and control groups and were also used to show the absolute difference in vaccination rates at registration between the experimental and control groups. Using multivariable logistic regression, we tested the impact of each of the incentive conditions on swab response rate by age, sex at birth, and area deprivation (relative response rate [RRR] with 95% CI). The reference group was the no-incentive condition—eg, the response rates for females in the £10 (US $12.5), £20 (US $25.0), and £30 (US $37.5) incentives groups were compared to females in the no-incentive group (£0 [US $0.0]). We tested interaction terms for age, sex at birth, and area deprivation by incentive (incentive*age, incentive*sex at birth, and incentive*IMD), which can be interpreted as testing whether the estimated effects of incentives on swab response rates differ by each of these 3 covariates.

## Results

### Overview

**Figure 2.**
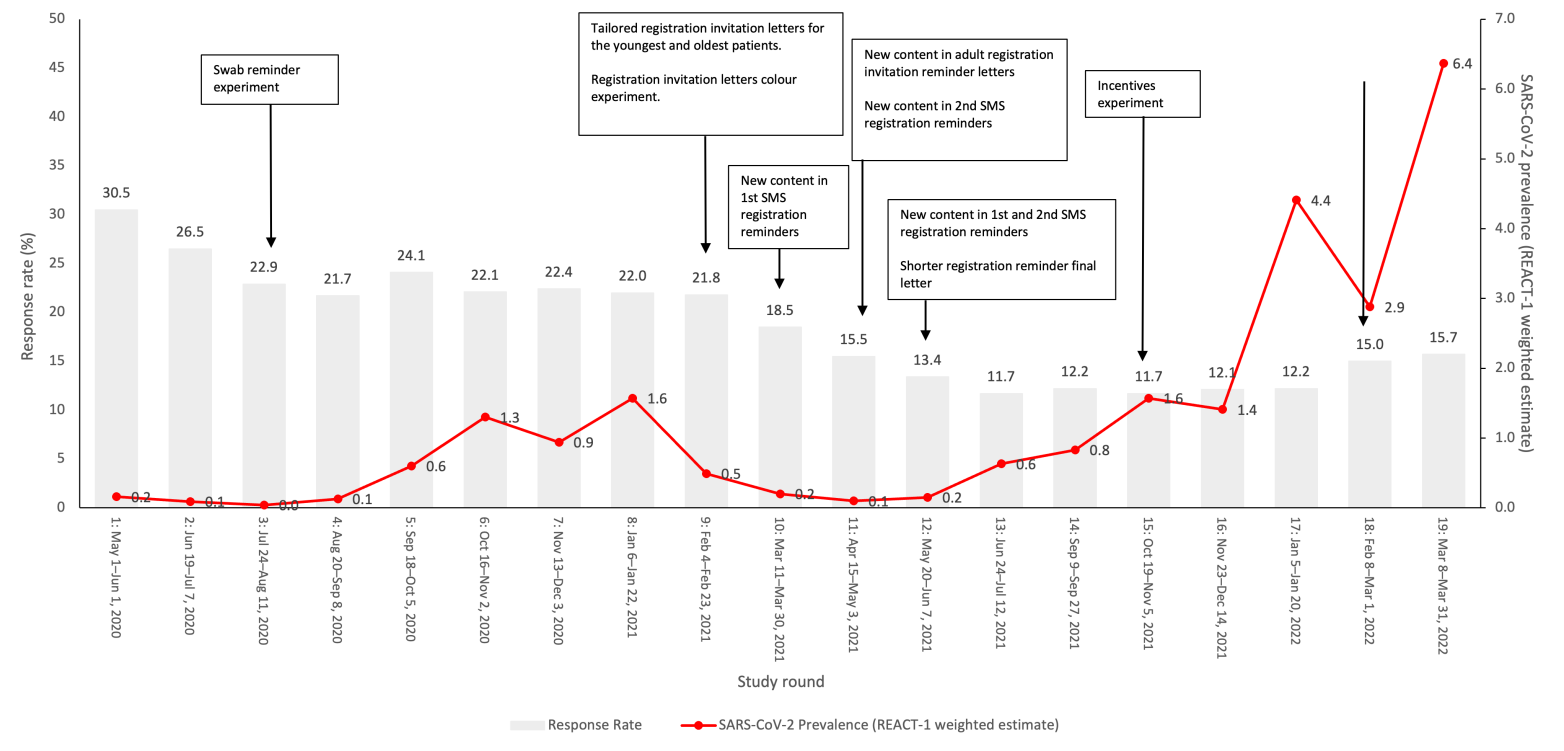
REACT-1 study timeline over 19 rounds of data collection showing response rates, SARS-CoV-2 prevalence (weighted), and timing of experiments to improve response. England, May 1, 2020 to March 31, 2022. REACT-1 Response Rate: number of valid swabs returned/number of invitations. We report weighted SARS-CoV-2 swab-positivity prevalence for individuals aged 5 years and older from all rounds of the REACT-1 study. REACT-1: REal-time Assessment of Community Transmission-1; SARS-CoV-2: Severe Acute Respiratory Syndrome CoronaVirus 2.

Overall, 24.2% (3,393,595/14,036,117) of invitees registered for the study, and 74% (2,512,797/3,393,595) of those registered returned valid swabs, giving an overall response rate for the REACT-1 study (number of valid swabs/number of invitations) of 17.9% (2,512,797/14,036,117) [19]. Whilst the rate at which registered participants returned valid swabs remained relatively stable across rounds (range 67.2%-78.9%), response rates varied more widely, ranging from 11.7% in rounds 13 (98,233/841,227) (June 24 to July 12, 2021) and 15 (100,112/859,184) (October 19 to November 5, 2021) to 30.5% in round 1 (120,620/395,020) (May 1 to June 1, 2020, during the first lockdown in England) (Figure 2). The following groups were relatively underrepresented: younger people, men, ethnic minorities, and those living in the most deprived areas (comparing achieved sample profiles with population profiles) (Table S1 in Multimedia Appendix 2).

### Swab Reminder and Tailored Letter or SMS Experiments

Table S2 in Multimedia Appendix 2 summarizes the results of the swab reminder and tailored letter or SMS experiments. Sending an additional reminder (email or SMS) to those who registered resulted in a small increase in response rate: those receiving a third swab reminder (experimental groups B and C) were more likely to return a completed swab compared to those receiving the standard Email-SMS approach (group B vs control: 73% vs 70.2%, percentage difference 2.8% [95% CI 1.9%-3.7%]; group C vs control 73.3% vs 70.2%, percentage difference 3.1% [95% CI 2.2%-4%]).

In round 9 (February 4-23, 2021), both experimental invitation letters A and B had a small but positive impact on response rate in participants aged ≥70 years of 0.9% (95% CI 0.2%-1.5%) and 1.2% (95% CI 0.6%-1.8%) percentage difference, respectively, compared to the standard invitation letter. For participants aged 5‐12 years, experiment letter C generated a slightly higher response rate compared to the standard letter (16.6% vs 15.9%; percentage difference 0.7% (95% CI 0.1%-1.4%)). In round 11 (April 15 to May 3, 2021) and round 12 (May 20 to June 7, 2021), the experimental invitation reminder letters had a small positive impact on response rate compared to the standard letters: round 11 (new content), 5.6% vs 5.4%, percentage difference 0.2% (95% CI 0%-0.3%); round 12 (shorter), 2.3% vs 1.6%, percentage difference 0.8% (95% CI 0.7%-0.8%). We saw no effect on response rate for any of the other nonmonetary strategies (Table S2 in Multimedia Appendix 2).

### Incentives Experiment

**Figure 3.**
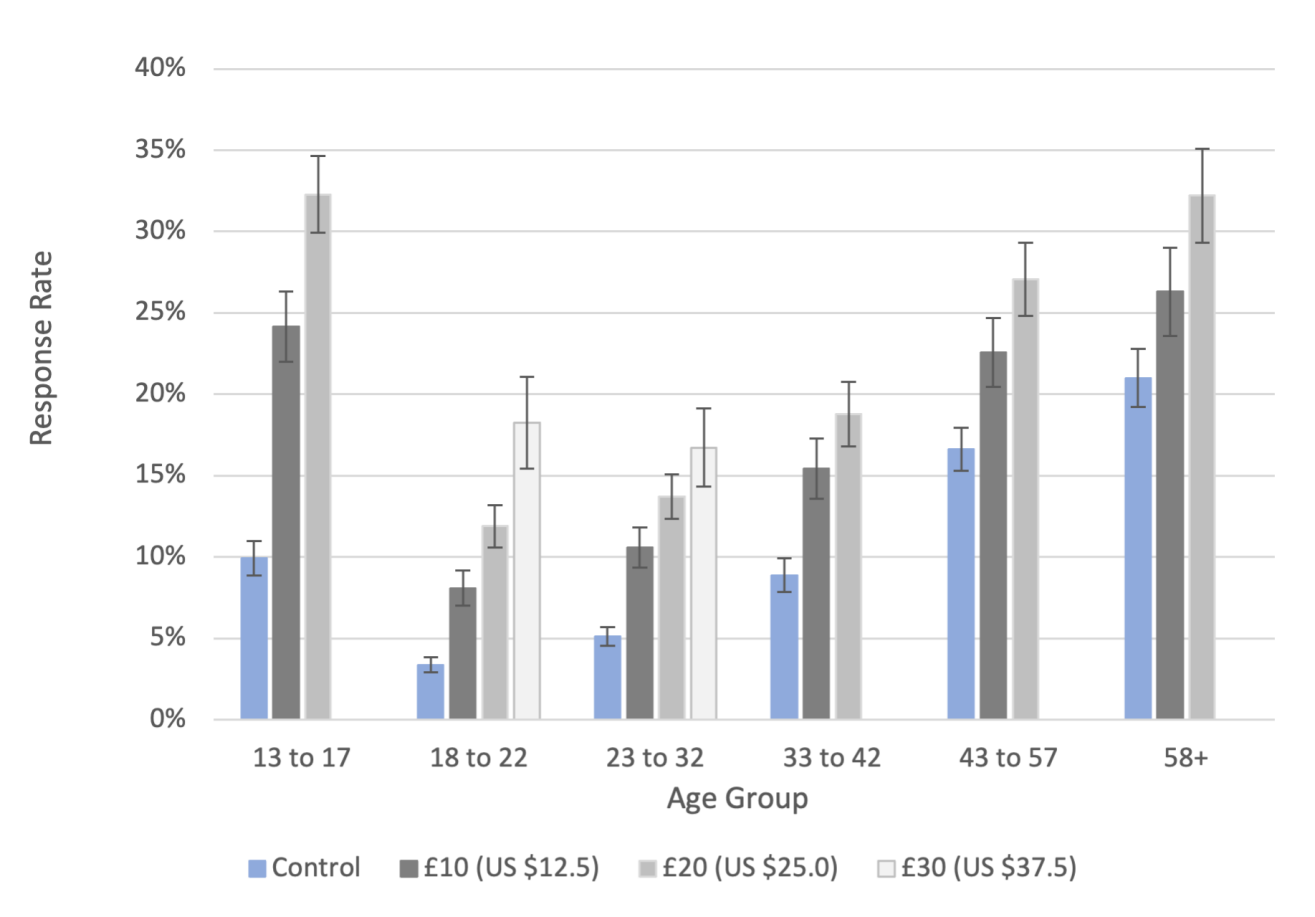
Swab response rates and 95% CIs for the intervention and control groups in the incentives experiment in round 15, England, October 19 to November 5, 2021. Note: Participants randomly allocated to experimental and control groups. (1) £10 (US $12.5) conditional incentive (n=10,900), (2) £20 (US $25.0) conditional incentive (n=10,900), (3) £30 (US $37.5) conditional incentive (only for 18- to 32-year-olds) (n=1750), and (4) control group (n=23,500).

The conditional monetary incentives increased the response rate across all age groups but were particularly effective among the lowest responding groups, those aged 13‐17 years and 18‐22 years (Figure 3 and Tables S3 and S4 in Multimedia Appendix 2). Table 3 shows the RRR for each incentive level by age, sex at birth, and area deprivation. The higher the monetary value of the incentive, the higher the response rate. For example, in participants aged 18‐22 years, the response rate in the control group was 3.4% (95% CI 2.9%-3.8%), increasing to 8.1% (95% CI 7.0%-9.2%), 11.9% (95% CI 10.6%-13.2%), and 18.2% (95% CI 15.4%-21.1%) with £10 (US $12.5), £20 (US $25.0), and £30 (US $37.5) incentives, respectively. The largest relative increase was with the £30 (US $37.5) incentive in 18‐ to 22-year-olds (RRR 5.4 [95% CI 4.4-6.7]) (Table 3). All incentive conditions led to a greater increase in response rate in younger age groups. The £20 (US $25.0) incentive led to a greater increase in the more deprived areas, RRR 2.7 (95% CI 2.2-3.3) for the most deprived quintile and RRR 1.8 (95% CI 1.6-2.1) for the least deprived.

**Table 3.** Variation in relative response rates (RRR) and 95% CI for the interventions compared to the control group by age, sex at birth, and area deprivation (IMD^a^) in the incentives experiment in round 15, England, October 19 to November 5, 2021.

	£10 (US $12.5)RRR (95% CI)	£20 (US $25.0)RRR (95% CI)	£30 (US $37.5)RRR (95% CI)
Age^b^ (years)			
13-17	2.4 (2.1-2.8)	3.3 (2.6-3.7)	N/A
18-22	2.4 (2.0-2.9)	3.5 (3.0-4.2)	5.4 (4.4-6.7)
23-32	2.1 (1.7-2.4)	2.7 (2.3-3.1)	3.3 (2.7-3.9)
33-42	1.7 (1.5-2.0)	2.1 (1.8-2.5)	—^c^
43-57	1.4 (1.2-1.5)	1.6 (1.5-1.8)	—
58+	1.3 (1.1-1.4)	1.5 (1.4-1.7)	—
*P* value for interaction between incentive and age	<.001	<.001	<.001
Sex at birth^b^^d^			
Male	1.4 (1.3-1.6)	1.8 (1.7-2.1)	3.7 (3.0-4.7)
Female	1.5 (1.4-1.6)	1.8 (1.6-2.0)	3.7 (3.1-4.4)
*P* value for interaction between incentive and sex at birth	.37	.96	.68
IMD^bd^			
1—most deprived	1.8 (1.5-2.3)	2.7 (2.2-3.3)	4.8 (3.3-7.0)
2	1.6 (1.3-1.9)	1.9 (1.6-2.3)	4.0 (2.9-5.5)
3	1.4 (1.2-1.6)	1.5 (1.3-1.8)	3.4 (2.5-4.8)
4	1.3 (1.1-1.5)	1.7 (1.5-1.9)	3.2 (2.4-4.3)
5—least deprived	1.5 (1.3-1.7)	1.8 (1.6-2.1)	3.5 (2.6-4.8)
*P* value for interaction between incentive and IMD	.38	.01	0.72

a IMD: Index of Multiple Deprivation

b Reference group, ie, the reference group for each row is the no incentive condition. For example, the RRR for female £10 (US $12.5), female £20 (US $25.0), and female £30 (US $37.5) is versus female £0 (US $0.0). *P* value for main effect of incentive on response rate for all row comparisons <.001.

c Not applicable

d Age-standardized weighting applied to calculate swab response rate with the control group totals used as the sample profiles.

**Figure 4.**
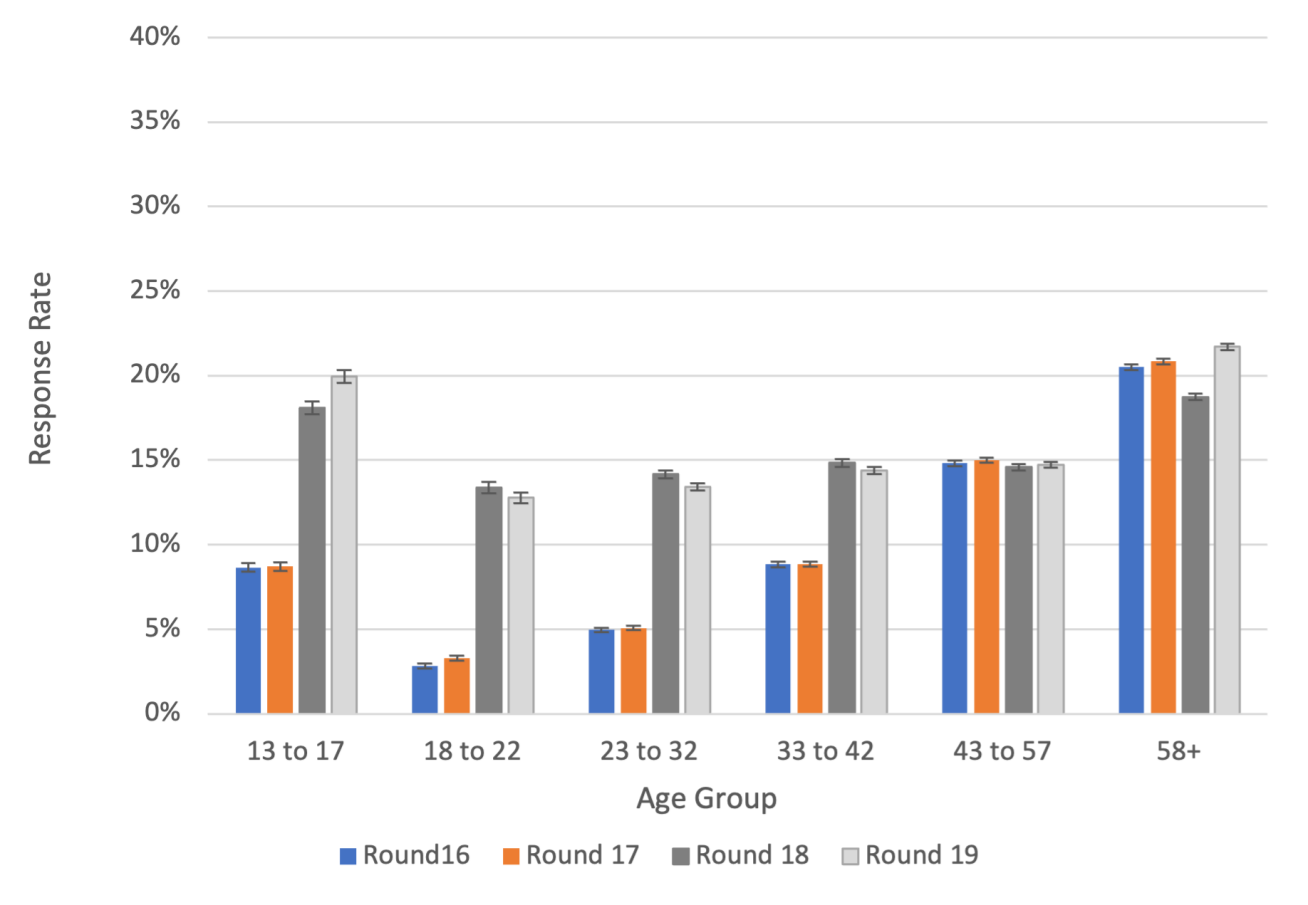
Swab response rates and 95% CIs for round 16 (November 23 to December 14, 2021), round 17 (January 5-20, 2022), and rounds 18 (February 8 to March 1, 2022) and 19 (March 8-31, 2022) in which incentives were used selectively, England. Note: Incentive amounts used in rounds 18 and 19: £10 (US $12.5) for 13- to 17-year-olds and 35- to 44-year-olds, £20 (US $25.0) for 18- to 34-year-olds, and no incentives for other age groups.

Following the results of the selective use of incentives in round 15, they were introduced in rounds 18 (Feb 8-Mar 1, 2022) and 19 (Mar 8-Mar 31, 2022). For returning their completed test, those aged 13‐17 and 35‐44 years were offered a gift voucher worth £10 (US $12.5), while those aged 18‐34 years were offered a voucher worth £20 (US $25.0). In these final 2 rounds, this had the effect of increasing the swab response rate in these groups and was associated with less variation in response rate by age (Figure 4), suggesting that the selective use of incentives reduced participation bias by age.

Table S5 in Multimedia Appendix 2 shows the effective sample sizes and sample efficiency for each round of REACT-1. The effective sample size measures the size of a (unweighted) simple random sample that would achieve the same precision (standard error) as the design used. The efficiency of a sample is given by the ratio of the effective sample size to the actual sample size. Rounds 18 and 19, where selective use of incentives was used, saw the fourth and second highest (respectively) effective sample sizes of any REACT-1 round, and the highest sample efficiency for any REACT-1 round.

Overall, vaccination rates were higher in REACT-1 participants than in the general population (Tables S6 and S7 in Multimedia Appendix 2). For example, by October 24, 2021, just over 3 quarters of 18‐ to 22-year-olds had received at least one vaccine dose nationally (Table S6 in Multimedia Appendix 2) [29], lower than the 84.0% (95% CI 78.1%-88.6%) in the round 15 (October 19-November 5, 2021) control group for that age (Table S7 in Multimedia Appendix 2). With the incentives that proportion declined to 82.1% (95% CI 76.0%-86.8%), 73.9% (95% CI 68.6%- 78.7%), and 75.9% (95% CI 67.9%-82.5%) for £10 (US $12.5), £20 (US $25.0), and £30 (US $37.5), respectively, suggesting that the selective use of incentives reduced participation bias in relation to vaccination status as a proxy for health behaviors.

## Discussion

### Principal Findings

In this large population-based study of the prevalence of SARS-CoV-2 infection in England, we tested several measures to increase response rates and reduce nonresponder bias. We found that changes to the wording of letters, timing, and numbers of reminders made only limited differences to response rates, with a maximum increase in response rate of 3.1 percentage points for additional swab reminders sent to people who had already registered for the study. Sending an additional reminder, regardless of its form (SMS or email), increased response. This is consistent with other studies in the literature [30]. These reminder strategies may have helped slow, but did not halt, the decline in response rates over time observed during REACT-1. Nonetheless, these findings informed the swab reminder strategy and invitation letter wording in later rounds. In contrast, the offer of a financial incentive conditional on the return of a completed swab made a more substantial difference of up to 22.3 percentage points and was particularly effective in those with a lower propensity to respond: younger age groups and those living in more deprived areas. Similarly, incentives increased the return of completed swabs by unvaccinated individuals so that COVID-19 vaccination rates were more in keeping with those in the general population at the time. Thus, the selective use of incentives may reduce nonresponder bias in relation to factors of interest in population health research beyond sociodemographic characteristics.

The selective use of incentives was subsequently adopted from round 18, making the achieved sample more representative by age, with a reduction in age-based variation in response rates. Previous research suggests that ethnic minorities [31], individuals living in more deprived areas [32], those in urban areas [33], and the youngest and oldest age groups [34,35] are the least likely to respond in general population surveys. Using incentives selectively allowed us, at modest cost, to increase recruitment among such groups and hence increase the effective sample size; thus, in round 18, the effective sample size was over 10,000 greater compared to round 17, even though we received circa 7000 fewer swabs. We were able to reduce the number of invitations sent out while achieving a similar number of completed swabs returned as in earlier rounds when response rates were higher.

Using incentives selectively has been tried in UK social surveys previously and is common practice in the United States, where studies show they are cost-effective, improve response, and reduce bias [11,13,23,36]. From an ethical perspective, in the selective use of (versus universal) incentives, it was important to consider not only issues of equity but also cost and the public interest in continuing to obtain high-quality data, covering all sectors of society, to monitor the spread of a serious disease. This needs to be balanced against the possible disappointment of some participants who learn others are being offered a (larger) incentive. These considerations might apply to many population-based surveys. We accept that the argument for using incentives selectively may have been more persuasive in the context of REACT-1, a study to measure the spread of SARS-CoV-2 during the pandemic, the policy responses to which had far-reaching consequences for the way of life of every person in England.

Both unconditional and conditional financial incentives have been shown to significantly increase response rates to both postal and web-based surveys [37,38]. Although unconditional incentives appear to have the largest effect, the conditional approach is more cost-effective [37,38]. Unconditional incentives have been used in social surveys in the United Kingdom, and in experiments in how to increase response rates [39,40]. Unconditional incentives were not an option for REACT-1 due to the constraints of the survey budget.

### Limitations

In terms of limitations, it was not possible to ascertain the extent to which noncontact (ie, the intended recipient did not receive the invitation letter) accounted for nonresponse. Such situational factors, for example, not informing their general practitioner of a change in address or having moved with no forwarding address (shown to be greater for young adults and lower socioeconomic groups) [32] will not be affected by the experimental conditions; therefore, our estimates of effect are likely conservative, as invitations sent out do not necessarily mean that invitations were received. In addition, the unique circumstances of carrying out such assessments of response rates during a global pandemic may not “read across” to other less pressing issues.

### Conclusions

We achieved small improvements in response rates by varying the number, order, and content of invitations and reminders but much larger effects were seen through the use of monetary incentives. Lessons learnt from the REACT-1 study may help inform the design and implementation of future population-based surveys where the intent is to obtain as representative a sample as possible and to reduce nonresponse bias at reasonable cost. The results suggest selectively using incentives with younger and more deprived individuals may be justifiable to achieve these ends.

## Supplementary material

10.2196/60022 Multimedia Appendix 1 Supplementary methods.

10.2196/60022 Multimedia Appendix 2 Supplementary tables.

## Abbreviations

Ct: cycle threshold
IMD: Index of Multiple Deprivation
NHS: National Health Service
REACT-1: Real-time Assessment of Community Transmission-1
RRR: relative response rate
rt-PCR: reverse transcriptase polymerase chain reaction
