# Supplementary material for: Strategies to Increase Response Rate and Reduce Nonresponse Bias in Population Health Research: Analysis of a Series of Randomized Controlled Experiments during a Large COVID-19 Study
Source: JMIR Public Health Surveill. 2025 Jan 9;11:e60022. doi: 10.2196/60022 (PMC11737284; doi:10.2196/60022)
Supplement: Multimedia Appendix 1 [file publichealth-v11-e60022-s001.docx]

**Multimedia Appendix 1: Supplementary Methods**

**Tailored letter/SMS experiments**

The registration invitation letters were designed to be informative about the purpose of the study, and to give clear instructions on the steps to register. Invitation letters varied by different sample groups (adults aged 18 years or over, parents or guardians of sampled children aged 5 to 12 years, parents and guardians of sampled children aged 13 to 17 years, and children aged 13 to 17 years). Parents of sampled children aged 13 to 17 years received an invitation letter addressed to them, asking them to pass on an enclosed letter addressed to their sampled child.

*Round 9: Tailored registration invitation letters for the youngest and oldest patients*

Some experiment letters sent to those aged 70 years or over had an additional focus on the importance of participating to measure the spread of the virus among that age group, even if they had been offered vaccination. Others had an additional focus on the vulnerability of that age group to COVID-19.

At the time, schools were preparing to undertake “mass testing” programmes using lateral flow test devices, so it was important to communicate that those aged 5-12 years should participate even if they had recently taken a different test. Two experiment letters were tested, each emphasising this message in a different way.

Participants for each target age group were randomly allocated to the experimental conditions as set out in Table 2 in the main manuscript. Groups outside these target age groups were sent the standard letter.

*Round 9: Varying the use of colour in registration invitation letters*

We tested using red text in the registration invitation letters to improve response, as they might be seen as more important. Half of the sample were allocated to the red invitation letter group and half to the standard invitation letter group (which used blue text).

*Round 10: Including new content in SMS registration reminders*

New messaging was developed for the first SMS reminder (relevant to the current situation in the pandemic, and which had not been communicated previously), and the registration deadline specified in the second SMS reminder only (Table 2, main manuscript).

*Round 11: Including new content in adult registration invitation reminder letters*

New wording in the adult invitation reminder letters was tested in Round 11. One quarter of the sample received a reminder letter with new wording asking them to take a test to help prevent the spread of COVID-19 and explaining that taking part would help the Government work out the best way to manage the pandemic (Table 2, main manuscript). The letter also mentioned testing for new variants, that the study compared people who had been vaccinated with those who had not, and that taking part would help inform the vaccine strategy and help to avoid lockdowns. One quarter of the sample received the standard reminder letter used in Round 10, asking them to take part to help the Government plan how to keep them and others safe, to help measure how many people had COVID-19 and explaining that taking part would help the Government work out the best way to manage the pandemic (Table 2, main manuscript).

*Round 11: Including new content in the second SMS registration reminder*

A new variation of the second SMS registration reminder was compared to the standard messaging used in the second registration reminder SMS. The standard second SMS registration reminder mentioned the deadline for registering and that the study is closing soon, while the new variation mentioned monitoring infection rates and new variants of the virus (Table 2, main manuscript).

*Round 12: Including new content in the first and second SMS registration reminder*

As in other Rounds, the sample was randomly allocated to four batches to facilitate experiments.

For the experiment with the first SMS reminder, the first, third and fourth batches of sample were allocated to the standard first SMS reminder, and the second batch to new wording (Table 2, main manuscript). For the experiment with the second SMS reminder, the first and third batches of sample were allocated to the standard second SMS reminder, and the second and fourth batches of sample to new wording (Table 2, main manuscript).

*Round 12: Introducing a new shorter registration invitation final reminder letter*

An additional reminder letter was introduced as the final reminder letter at Round 12. A standard reminder letter (double sided) was tested against a single page succinct letter which also included a QR code participants could scan to enable easier completion of the registration survey on a mobile device. The experiment group (first, second and third batches of sample) were allocated the single page succinct letter, while the fourth batch of sample were allocated the standard reminder letter (Table 2, main manuscript).

**Sample size calculations for the incentive experiment**

The sample size estimates for the incentive experiment were calculated using the response rates observed at Round 13 for six age group bands. The aim was to detect a significant increase in the response rate with 80% power at the 5% significance level for the following percentage point (pp) increases in response rates compared to the control group (£0):

|  | **Minimum detectable percentage point (pp) difference for comparisons between incentive experiment conditions** | | |
| --- | --- | --- | --- |
|  | **£0 v £10/£20** | **£10 v £20** | **£0 v £30** |
| 13 to 17 | 2.95pp | 3.40pp |  |
| 18 to 22 | 1.55pp | 1.80pp | 3.00pp |
| 23 to 32 | 1.80pp | 2.10pp | 3.00pp |
| 33 to 42 | 2.85pp | 3.30pp |  |
| 43 to 57 | 3.25pp | 3.80pp |  |
| 58+ | 4.50pp | 5.10pp |  |
