# Supplementary material for: Strategies to Increase Response Rate and Reduce Nonresponse Bias in Population Health Research: Analysis of a Series of Randomized Controlled Experiments during a Large COVID-19 Study
Source: JMIR Public Health Surveill. 2025 Jan 9;11:e60022. doi: 10.2196/60022 (PMC11737284; doi:10.2196/60022)
Supplement: Multimedia Appendix 2 [file publichealth-v11-e60022-s002.docx]

**Multimedia Appendix 2: Supplementary Tables**

**Supplementary Table S1. Overall REACT-1 study response rate (number of valid swabs returned/number of invitations) by key sociodemographic characteristics across 19 rounds of data collection: England, May 1, 2020–March 31, 2022**

|  | **Rate Response** |
| --- | --- |
| **Overall** (2,512,797 valid swabs returned/14,036,117 invitations) | 17.9% |
| **Sex** |  |
| Female | 19.9% |
| Male | 16.1% |
| **Age (in years)** |  |
| 5 to 12 | 12.5% |
| 13 to 17 | 16.7% |
| 18 to 24 | 8.6% |
| 25 to 34 | 11.0% |
| 35 to 44 | 15.6% |
| 45 to 54 | 20.6% |
| 55 to 64 | 26.2% |
| 65 to 74 | 31.8% |
| 75 to 84 | 22.2% |
| 85+ | 8.9% |
| **Region** |  |
| North East | 16.9% |
| North West | 15.4% |
| Yorkshire and The Humber | 17.2% |
| East Midlands | 19.9% |
| West Midlands | 16.4% |
| East of England | 21.2% |
| London | 11.6% |
| South East | 22.7% |
| South West | 23.2% |
| **Index of Multiple Deprivation Decile** |  |
| 1 – most deprived | 8.0% |
| 2 | 10.0% |
| 3 | 12.2% |
| 4 | 14.9% |
| 5 | 17.8% |
| 6 | 20.1% |
| 7 | 22.2% |
| 8 | 23.6% |
| 9 | 25.7% |
| 10 - least deprived | 29.0% |

**Supplementary Table S2. Swab response rates for swab reminders and tailored registration invitation letters and SMS reminders experiments, Round 3 (Jul 24–Aug 11, 2020) and 9-12 (between Feb 4 and Jun 7, 2021)**

| **Round** | **Letter/SMS type** | **No. of swabs returned / No. of registrations** | **Response Rate** | **Absolute difference (95% CI)** | ***P* value** |
| --- | --- | --- | --- | --- | --- |
| **Round 3**^a^ | Control group | 7854 / 11,194 | 70.2% | - |  |
|  | Experimental group A | 7809 / 11,154 | 70.0% | -0.2 (-1.4, 1.0) | .80 |
|  | Experimental group B | 70,296 / 96,335 | 73.0% | 2.8 (1.9, 3.7) | <.001 |
|  | Experimental group C | 70,547 / 96,305 | 73.3% | 3.1 (2.2, 4.0) | <.001 |
|  |  | **No. of swabs returned / No. of invitations** |  |  |  |
| **Round 9**^b^  **≥70 years** | Standard Adult invitation letter | 9070 / 37,037 | 24.5% | - |  |
|  | Experiment invitation letter A | 9409 / 37,037 | 25.4% | 0.9 (0.2, 1.5) | .004 |
|  | Experiment invitation letter B | 9509 / 37,036 | 25.7% | 1.2 (0.6, 1.8) | <.001 |
| **Round 9**^b^  **5-12 years** | Standard Child (5-12 years) invitation letter | 3810 / 24,009 | 15.9% | - |  |
|  | Experiment invitation letter C | 3981 / 24,009 | 16.6% | 0.7 (0.1, 1.4) | .03 |
|  | Experiment invitation letter D | 3950 / 24,008 | 16.5% | 0.6 (0.1, 1.2) | .08 |
|  |  | **No. of swabs returned / No. of invitations not yet registered after initial invite** |  |  |  |
| **Round 9**^c^ | Standard registration reminder letter | 20,712 / 306,012 | 6.8% | - |  |
|  | Experiment registration reminder letter E | 20,376 / 305,041 | 6.7% | -0.1 (-0.2, 0.0) | .17 |
| **Round 10**^d^ | Standard SMS first registration reminder | 2500 / 50,000 | 5.0% | - |  |
|  | Experiment first SMS registration reminder | 22,220 / 480,283 | 5.2% | 0.2 (0.0, 0.4) | .12 |
| **Round 11**^e^ **(Adults)** | Standard invitation reminder letter | 9625 / 178,828 | 5.4% | - |  |
|  | Experiment invitation reminder letter A | 9952 / 178,809 | 5.6% | 0.2 (0.0, 0.3) | .02 |
| **Round 12**^f^ | Standard SMS first registration reminder | 31,290 / 321,042 | 9.7% | - |  |
|  | Experiment first SMS registration reminder | 12,265 / 155,683 | 7.9% | -1.9 (-2.0, -1.7) | <.001 |
|  |  | **No. of swabs returned / No. of invitations not yet registered after first reminder** |  |  |  |
| **Round 11**^g^ | Standard SMS second registration reminder | 1957 / 127,028 | 1.5% |  |  |
|  | Experiment second SMS registration reminder | 1986 / 127,028 | 1.6% | 0.0 (-0.1, 0.1) | .64 |
| **Round 12**^h^ | Standard invitation final reminder letter | 2644 / 169,845 | 1.6% | - |  |
|  | Shorter invitation final reminder letter | 7947 / 342,191 | 2.3% | 0.8 (0.7, 0.8) | <.001 |
| **Round 12**^i^ | Standard SMS second registration reminder | 1200 / 272,836 | 0.4% | - |  |
|  | Experiment second SMS registration reminder | 543 / 136,026 | 0.4% | 0.0 (-0.0, 0.1) | .06 |

a New swab reminder Email/SMS sequencing, Round 3; b Tailored registration invitation letters for the youngest and oldest invitees, Round 9; c Different colour used for registration reminder letters for all invitees, Round 9; d New content in SMS registration reminder, Round 10; e New content for adult invitation letter reminder letter, Round 11; f New content in first SMS registration reminder, Round 12; g New content in second SMS registration reminder, Round 11; h New shorter registration invitation final reminder letter, Round 12; i New content in second SMS registration reminder, Round 12.

Note: Confidence Interval = CI

**Supplementary Table S3. Characteristics of the Round 15 (Oct 19–Nov 5, 2021) study population by i) number of invitations sent, ii) number of swabs returned and swab response rates for the intervention and control groups in the incentives experiment**

|  | **No. of Invitations Sent** | | | **No. of Swabs Returned** | | | **Response Rate** | | | **Absolute difference (95% CI)** | |
| --- | --- | --- | --- | --- | --- | --- | --- | --- | --- | --- | --- |
|  | **Control** | **£10** | **£20** | **Control** | **£10** | **£20** | **Control** | **£10** | **£20** | **£10** | **£20** |
| **Age** |  |  |  |  |  |  |  |  |  |  |  |
| 5 to 12 | 0 | 0 | 0 | 0 | 0 | 0 | N/A | N/A | N/A | N/A | N/A |
| 13 to17 | 3031 | 1512 | 1512 | 301 | 365 | 488 | 9.9% | 24.1% | 32.3% | 14.2  (12.1, 16.4) | 22.3  (20.0, 24.6) |
| 18 to 22 | 5588 | 2414 | 2423 | 188 | 195 | 288 | 3.4% | 8.1% | 11.9% | 4.7  (3.7, 5.7) | 8.5  (7.4, 9.6) |
| 23 to 32 | 5789 | 2449 | 2441 | 296 | 259 | 335 | 5.1% | 10.6% | 13.7% | 5.5  (4.3, 6.6) | 8.6  (7.4, 9.9) |
| 33 to 42 | 2995 | 1472 | 1480 | 266 | 227 | 278 | 8.9% | 15.4% | 18.8% | 6.5  (4.6, 8.5) | 9.9  (7.9, 11.9) |
| 43 to 57 | 3026 | 1516 | 1503 | 503 | 342 | 407 | 16.6% | 22.6% | 27.1% | 5.9  (3.5, 8.3) | 10.5  (8.0, 12.9) |
| 58+ | 2006 | 1015 | 1012 | 421 | 267 | 326 | 21.0% | 26.3% | 32.2% | 5.3  (2.2, 8.5) | 11.2  (8.0, 14.5) |
| **^a^Sex at birth** |  |  |  |  |  |  |  |  |  |  |  |
| Male | 11205 | 5173 | 5151 | 1354 | 896 | 1152 | 12.1% | 17.3% | 22.4% | 5.2  (3.5, 6.9) | 10.3  (8.5, 12.1) |
| Female | 11230 | 5202 | 5220 | 1659 | 1142 | 1377 | 14.8% | 22.0% | 26.4% | 7.2  (5.4, 9.0) | 11.6  (9.7, 13.5) |
| **^a^IMD** |  |  |  |  |  |  |  |  |  |  |  |
| 1 most deprived | 4441 | 2151 | 2040 | 270 | 238 | 333 | 6.1% | 11.1% | 16.3% | 5.0  (3.0, 6.9) | 10.3  (8.0, 12.5) |
| 2 | 4635 | 2074 | 2160 | 456 | 328 | 405 | 9.8% | 15.8% | 18.7% | 6.0  (3.5, 8.4) | 8.9  (6.3, 11.5) |
| 3 | 4667 | 2035 | 2085 | 708 | 432 | 482 | 15.2% | 21.2% | 23.1% | 6.1  (3.2, 8.9) | 8.0  (5.1, 10.9) |
| 4 | 4412 | 2061 | 2002 | 775 | 471 | 594 | 17.6% | 22.9% | 29.7% | 5.3  (2.3, 8.3) | 12.1  (8.9, 15.3) |
| 5 least deprived | 4279 | 2057 | 2084 | 804 | 573 | 715 | 18.8% | 27.8% | 34.3% | 9.0  (5.8, 12.3) | 15.5  (12.2, 18.9) |

a age-standardised weighting applied to calculate swab response rate with the control group totals used as the sample profiles. Note: Confidence Interval = CI

**Supplementary Table S4. Characteristics of the Round 15 (Oct 19–Nov 5, 2021) study population aged 18-32 years by i) number of invitations sent, ii) number of swabs returned and swab response rates for the intervention and control groups in the incentives experiment**

|  | **No. of Invitations Sent** | | | | **No. of Swabs Returned** | | | | **Response Rate** | | | | **Absolute difference (95% CI)** | | |
| --- | --- | --- | --- | --- | --- | --- | --- | --- | --- | --- | --- | --- | --- | --- | --- |
|  | **Control** | **£10** | **£20** | **£30** | **Control** | **£10** | **£20** | **£30** | **Control** | **£10** | **£20** | **£30** | **£10** | **£20** | **£30** |
| **Age** |  |  |  |  |  |  |  |  |  |  |  |  |  |  |  |
| 18 to 22 | 5588 | 2414 | 2423 | 729 | 188 | 195 | 288 | 133 | 3.4% | 8.1% | 11.9% | 18.2% | 4.7  (3.7, 5.7) | 8.5  (7.4, 9.6) | 14.9  (13.2, 16.5) |
| 23 to 32 | 5789 | 2449 | 2441 | 921 | 296 | 259 | 335 | 154 | 5.1% | 10.6% | 13.7% | 16.7% | 5.5  (4.3, 6.6) | 8.6  (7.4, 9.9) | 11.6  (9.9, 13.3) |
| **^a^Sex at birth** |  |  |  |  |  |  |  |  |  |  |  |  |  |  |  |
| Male | 5739 | 2436 | 2417 | 816 | 206 | 174 | 229 | 110 | 3.6% | 7.1% | 9.5% | 13.5% | 3.5  (2.3, 4.8) | 5.9  (4.5, 7.3) | 9.9  (7.4, 12.4) |
| Female | 5638 | 2427 | 2447 | 833 | 319 | 306 | 413 | 173 | 5.7% | 12.6% | 16.9% | 20.8% | 7.0  (5.4, 8.6) | 11.2  (9.5, 13) | 15.2  (12.2, 18.1) |
| **^a^IMD** |  |  |  |  |  |  |  |  |  |  |  |  |  |  |  |
| 1 most deprived | 2647 | 1132 | 1181 | 357 | 73 | 76 | 118 | 48 | 2.8% | 6.7% | 10.0% | 13.3% | 4.0  (2.2, 5.7) | 7.3  (5.3, 9.3) | 10.6  (6.9, 14.2) |
| 2 | 2706 | 1107 | 1143 | 386 | 107 | 86 | 114 | 60 | 3.9% | 7.7% | 9.9% | 15.6% | 3.8  (1.9, 5.7) | 6.0  (3.9, 8.1) | 11.7  (7.8, 15.6) |
| 3 | 2264 | 1002 | 969 | 334 | 100 | 119 | 128 | 51 | 4.4% | 11.8% | 13.2% | 15.2% | 7.4  (5.0, 9.8) | 8.8  (6.2, 11.3) | 10.7  (6.7, 14.8) |
| 4 | 2006 | 857 | 839 | 322 | 133 | 94 | 140 | 69 | 6.6% | 11.0% | 16.7% | 21.3% | 4.3  (1.7, 6.9) | 10.0  (7.0, 13) | 14.7  (9.7, 19.6) |
| 5 least deprived | 1754 | 765 | 732 | 251 | 112 | 105 | 143 | 56 | 6.4% | 13.7% | 19.5% | 22.3% | 7.4  (4.4, 10.3) | 13.1  (9.8, 16.5) | 15.9  (10.5, 21.4) |

a age-standardised weighting applied to calculate swab response rate with the control group totals used as the sample profiles. Note: Confidence Interval = CI

**Supplementary Table S5.** **Effective sample sizes and sample efficiency for each Round of REACT-1**

| 1. **Round** | 1. **Prevalence** | 1. **SE (CS)** | 1. **Low (95 CI)** | 1. **High (95 CI)** | 1. **DEFF** | 1. **Actual *n*** | 1. **Effective *n*** | 1. **Efficiency** |
| --- | --- | --- | --- | --- | --- | --- | --- | --- |
| 1. 1: May 1–Jun 1, 2020 | 1. 0.53% | 1. 0.03% | 1. 0.48% | 1. 0.59% | 1. 1.883 | 1. 121,629 | 1. 64,579 | 1. 53.1% |
| 1. 2: Jun 19–Jul 7, 2020 | 1. 0.09% | 1. 0.01% | 1. 0.07% | 1. 0.11% | 1. 1.957 | 1. 160,788 | 1. 82,171 | 1. 51.1% |
| 1. 3: Jul 24–Aug 11, 2020 | 1. 0.04% | 1. 0.01% | 1. 0.03% | 1. 0.05% | 1. 1.828 | 1. 163,313 | 1. 89,346 | 1. 54.7% |
| 1. 4: Aug 20–Sep 8, 2020 | 1. 0.13% | 1. 0.01% | 1. 0.10% | 1. 0.16% | 1. 2.703 | 1. 154,814 | 1. 57,284 | 1. 37.0% |
| 1. 5: Sep 18–Oct 5, 2020 | 1. 0.59% | 1. 0.03% | 1. 0.54% | 1. 0.65% | 1. 2.526 | 1. 175,887 | 1. 69,640 | 1. 39.6% |
| 1. 6: Oct 16–Nov 2, 2020 | 1. 1.29% | 1. 0.05% | 1. 1.20% | 1. 1.38% | 1. 2.659 | 1. 161,415 | 1. 60,695 | 1. 37.6% |
| 1. 7: Nov 13–Dec 3, 2020 | 1. 0.93% | 1. 0.04% | 1. 0.86% | 1. 1.00% | 1. 2.308 | 1. 169,126 | 1. 73,279 | 1. 43.3% |
| 1. 8: Jan 6–Jan 22, 2021 | 1. 1.56% | 1. 0.04% | 1. 1.47% | 1. 1.64% | 1. 2.087 | 1. 168,989 | 1. 80,972 | 1. 47.9% |
| 1. 9: Feb 4–Feb 23, 2021 | 1. 0.49% | 1. 0.03% | 1. 0.44% | 1. 0.54% | 1. 2.276 | 1. 165,909 | 1. 72,907 | 1. 43.9% |
| 1. 10: Mar 11–Mar 30, 2021 | 1. 0.20% | 1. 0.02% | 1. 0.17% | 1. 0.23% | 1. 1.969 | 1. 142,053 | 1. 72,151 | 1. 50.8% |
| 1. 11: Apr 15–May 3, 2021 | 1. 0.10% | 1. 0.01% | 1. 0.08% | 1. 0.13% | 1. 2.037 | 1. 127,857 | 1. 62,778 | 1. 49.1% |
| 1. 12: May 20–Jun 7, 2021 | 1. 0.15% | 1. 0.01% | 1. 0.12% | 1. 0.18% | 1. 1.560 | 1. 109,289 | 1. 70,054 | 1. 64.1% |
| 1. 13: Jun 24–Jul 12, 2021 | 1. 0.63% | 1. 0.03% | 1. 0.57% | 1. 0.69% | 1. 1.528 | 1. 98,796 | 1. 64,668 | 1. 65.5% |
| 1. 14: Sep 9–Sep 27, 2021 | 1. 0.81% | 1. 0.03% | 1. 0.75% | 1. 0.88% | 1. 1.363 | 1. 100,807 | 1. 73,967 | 1. 73.4% |
| 1. 15: Oct 19–Nov 5, 2021 | 1. 1.54% | 1. 0.05% | 1. 1.45% | 1. 1.63% | 1. 1.389 | 1. 100,709 | 1. 72,495 | 1. 72.0% |
| 1. 16: Nov 23–Dec 14, 2021 | 1. 1.40% | 1. 0.05% | 1. 1.31% | 1. 1.50% | 1. 1.520 | 1. 97,474 | 1. 64,132 | 1. 65.8% |
| 1. 17: Jan 5–Jan 20, 2022 | 1. 4.42% | 1. 0.08% | 1. 4.27% | 1. 4.58% | 1. 1.489 | 1. 102,279 | 1. 68,700 | 1. 67.2% |
| 1. 18: Feb 8–Mar 1, 2022 | 1. 2.89% | 1. 0.06% | 1. 2.77% | 1. 3.00% | 1. 1.205 | 1. 95,207 | 1. 79,025 | 1. 83.0% |
| 1. 19: Mar 8–Mar 31, 2022 | 1. 6.34% | 1. 0.08% | 1. 6.18% | 1. 6.50% | 1. 1.232 | 1. 109,459 | 1. 88,847 | 1. 81.2% |

Note: SE(CS) = Standard Error (Complex Samples); DEFF= design effect

**Supplementary Table S6. Vaccination uptake percentages (% who have had at least 1 dose) for COVID-19 by age group in England up to 24^th^ October 2021**

|  | **Vaccination Rate (% who have had at least 1 dose** |
| --- | --- |
| **Age (in years)** |  |
| 18 to 24 | 75.1% |
| 25 to 29 | 76.9% |
| 30 to 34 | 84.1% |
| 35 to 39 | 86.8% |
| 40 to 44 | 92.2% |
| 45 to 49 | 89.6% |
| 50 to 54 | 94.5% |
| 55 to 59 | 97.2% |
| 60 to 64 | 99.3% |
| 65 to 69 | 96.8% |
| 70 to 74 | 96.1% |
| 75 to 79 | 100% |
| 80+ | 95.3% |

Source: National Immunisation Management System (NIMS): https://www.england.nhs.uk/statistics/statistical-work-areas/covid-19-vaccinations/covid-19-vaccinations-archive/

**Supplementary Table S7. Vaccination rates at registration for the intervention and control groups in the incentives experiment in Round 15 (Oct 19–Nov 5, 2021)**

|  | **No. of vaccinated / Total no.** | | | | **Vaccination Rate (95% CI)** | | | | **Absolute difference (95% CI)** | | | ***P* value** | | |
| --- | --- | --- | --- | --- | --- | --- | --- | --- | --- | --- | --- | --- | --- | --- |
|  | **Control** | **£10** | **£20** | **£30** | **Control** | **£10** | **£20** | **£30** | **£10** | **£20** | **£30** | **£10** | **£20** | **£30** |
| **Age (in years)** |  |  |  |  |  |  |  |  |  |  |  |  |  |  |
| 5 to 12 | N/A | N/A | N/A | N/A | N/A | N/A | N/A | N/A | N/A | N/A | N/A | N/A | N/A | N/A |
| 13 to 17 | 119/301 | 132/365 | 182/488 | N/A | 39.5%  (34.2, 45.2) | 36.2%  (31.4, 41.2) | 37.3%  (33.1, 41.7) | N/A | -3.4  (-10.8, 4.0) | -2.2  (-9.2, 4.8) | N/A | .37 | .53 | N/A |
| 18 to 22 | 158/188 | 160/195 | 213/288 | 101/133 | 84.0%  (78.1, 88.6) | 82.1%  (76.0, 86.8) | 73.9%  (68.6, 78.7) | 75.9%  (67.9, 82.5) | -2.0  (-9.5, 5.5) | -10.1  (-17.4, -2.8) | -8.1  (-17.1, 0.85) | .60 | .01 | .07 |
| 23 to 32 | 237/296 | 206/259 | 268/335 | 125/154 | 80.1%  (75.1, 84.2) | 79.5%  (74.2, 84.0) | 80.0%  (75.4, 84.0) | 81.2%  (74.2, 86.6) | -0.53  (-7.2, 6.2) | -0.07  (-6.3, 6.2) | 1.1  (-6.6, 8.8) | .88 | .98 | .78 |
| 33 to 42 | 228/266 | 188/227 | 231/278 | N/A | 85.7%  (81.0, 89.4) | 82.8%  (77.3, 87.2) | 83.1%  (78.2, 87.1) | N/A | -2.9  (-9.4, 3.6) | -2.6  (-8.7, 3.5) | N/A | .38 | .40 | N/A |
| 43 to 57 | 433/503 | 296/342 | 349/407 | N/A | 86.1%  (82.8, 88.9) | 86.6%  82.5, 89.8) | 85.8%  (82.0, 88.8) | N/A | 0.47  (-4.2, 5.2) | -0.33  (-4.9, 4.2) | N/A | .85 | .89 | N/A |
| 58+ | 380/421 | 237/267 | 283/326 | N/A | 90.3%  (87.0, 92.8) | 88.8%  (84.4, 92.0) | 86.8%  (82.7, 90.1) | N/A | -1.5  (-6.2, 3.2) | -3.5  (-8.1, 1.2) | N/A | .53 | .14 | N/A |

Note: Confidence Interval = CI

**Supplementary Table S8.** **Swab response rates and 95% Confidence Intervals (CI) for the intervention and control groups in the incentives experiment in Round 15 (Oct 19–Nov 5, 2021), and Rounds 18 (Feb 8–Mar 1, 2022) and 19 (Mar 8–Mar 31, 2022) with targeted incentives approach**

Incentive amounts used in Rounds 18 and 19: (£10 for 13 to 17 year olds, £20 for 18 to 32 year olds, £30 for 33 to 42 year olds, no incentives for other age groups).

|  | **Response Rate (95% CI)** | | | | |
| --- | --- | --- | --- | --- | --- |
| **Age (in years)** | **Control** | **£10** | **£20** | **£30** |  |
| **Round 15** |  |  |  |  |  |
| 13 to 17 | 9.9% (8.9, 11.0) | 24.1% (22.0, 26.3) | 32.3% (29.9, 34.6) | N/A |  |
| 18 to 22 | 3.4% (2.9, 3.8) | 8.1% (7.0, 9.2) | 11.9% (10.6, 13.2) | 18.2% (15.4, 21.1) |  |
| 23 to 32 | 5.1% (4.5, 5.7) | 10.6% (9.4, 11.8) | 13.7% (12.4, 15.1) | 16.7% (14.3, 19.1) |  |
| 33 to 42 | 8.9% (7.9, 9.9) | 15.4% (13.6, 17.3) | 18.8% (16.8, 20.8) | N/A |  |
| 43 to 57 | 16.6% (15.3, 17.9) | 22.6% (20.5, 24.7) | 27.1% (24.8, 29.3) | N/A |  |
| 58+ | 21.0% (19.2, 22.8) | 26.3% (23.6, 29.0) | 32.2% (29.3, 35.1) | N/A |  |
|  |  |  |  |  |  |
| **Round 16** |  |  |  |  | **All** |
| 13 to 17 |  |  |  |  | 8.7% (8.4, 8.9) |
| 18 to 22 |  |  |  |  | 2.8% (2.7, 3.0) |
| 23 to 32 |  |  |  |  | 5.0% (4.8, 5.1) |
| 33 to 42 |  |  |  |  | 8.8% (8.7, 9.0) |
| 43 to 57 |  |  |  |  | 14.8% (14.7, 15.0) |
| 58+ |  |  |  |  | 20.5% (20.3, 20.7) |
| **Round 17** |  |  |  |  |  |
| 13 to 17 |  |  |  |  | 8.7% (8.5, 9.0) |
| 18 to 22 |  |  |  |  | 3.3% (3.1, 3.5) |
| 23 to 32 |  |  |  |  | 5.1% (5.0, 5.2) |
| 33 to 42 |  |  |  |  | 8.8% (8.7, 9.0) |
| 43 to 57 |  |  |  |  | 15.0% (14.8, 15.2) |
| 58+ |  |  |  |  | 20.8% (20.7, 21.0) |
| **Round 18** |  |  |  |  |  |
| 13 to 17 |  |  |  |  | 18.1% (17.7, 18.5) |
| 18 to 22 |  |  |  |  | 13.4% (13.0, 13.7) |
| 23 to 32 |  |  |  |  | 14.2% (13.9, 14.4) |
| 33 to 42 |  |  |  |  | 14.8% (14.6, 15.0) |
| 43 to 57 |  |  |  |  | 14.6% (14.4, 14.8) |
| 58+ |  |  |  |  | 18.7% (18.5, 18.9) |
| **Round 19** |  |  |  |  |  |
| 13 to 17 |  |  |  |  | 19.9% (19.5, 20.3) |
| 18 to 22 |  |  |  |  | 12.8% (12.5, 13.1) |
| 23 to 32 |  |  |  |  | 13.4% (13.2, 13.6) |
| 33 to 42 |  |  |  |  | 14.4% (14.2, 14.6) |
| 43 to 57 |  |  |  |  | 14.7% (14.5, 14.9) |
| 58+ |  |  |  |  | 21.7% (21.5, 21.9) |
